# Phylogeography of cultivated and wild *ophiopogon japonicus* based on chloroplast DNA: exploration of the origin and sustainable cultivation

**DOI:** 10.1186/s12870-023-04247-2

**Published:** 2023-05-08

**Authors:** Lu-ying Zhao, Yu-ling Liu, Yi Shen, Qiao-yan Zhang, Sha Liu, Qiu-ru Ren, Lu-ping Qin, Yi-qi Sun

**Affiliations:** College of Pharmaceutical Sciences, Zhejiang Chinese Medical University, Hangzhou, Zhejiang China

**Keywords:** Phylogeography, Origin, Domestication, *Ophiopogon japonicus*, Conservation

## Abstract

**Background:**

*Ophiopogon japonicus*, mainly planted in Sichuan (CMD) and Zhejiang (ZMD) province in China, has a lengthy cultivation history. During the long period of domestication, the genetic diversity of cultivated *O. japonicus* has substantially declined, which will affect the population continuity and evolutionary potential of this species. Therefore, it is necessary to clarify the phylogeography of cultivated *O. japonicus* to establish a theoretical basis for the utilization and conservation of the genetic resources of *O. japonicus*.

**Result:**

The genetic diversity and population structure of 266 *O. japonicus* individual plants from 23 sampling sites were analyzed based on 4 chloroplast DNA sequences (*atpB-rbcL*, *rpl16*, *psbA-trnH* and *rpl20-5’rps12*) to identify the effects of domestication on genetic diversity of cultivars and determine their geographic origins. The results showed that cultivated *O. japonicus* and wild *O. japonicus* had 4 and 15 haplotypes respectively. The genetic diversity of two cultivars (*H*_d_ = 0.35700, *π* = 0.06667) was much lower than that of the wild populations (*H*_d_ = 0.76200, *π* = 0.20378), and the level of genetic diversity in CMD (*H*_d_ = 0.01900, *π* = 0.00125) was lower than that in ZMD (*H*_d_ = 0.06900, *π* = 0.01096). There was significant difference in genetic differentiation between the cultivated and the wild (*F*_ST_ = 0.82044), especially between the two cultivars (*F*_ST_ = 0.98254). This species showed a pronounced phylogeographical structure (*N*_ST_ > *G*_ST_, *P* < 0.05). The phylogenetic tree showed that the genetic difference between CMD and ZMD was not enough to distinguish the cultivars between the two producing areas by using *O. amblyphyllus* Wang et Dai as an outgroup. In addition, both CMD and ZMD have a closer relationship with wild populations in Sichuan than that in Zhejiang. The results of the TCS network and species distribution model suggested that the wild population TQ located in Sichuan province could serve as the ancestor of cultivated *O. japonicus*, which was supported by RASP analysis.

**Conclusion:**

These results suggest that cultivated *O. japonicus* has experienced dramatic loss of genetic diversity under anthropogenic influence. The genetic differentiation between CMD and ZMD is likely to be influenced by founder effect and strong artificial selection for plant traits. It appears that wild populations in Sichuan area are involved in the origin of not only CMD but also ZMD. In addition, we also raise some suggestions for planning scientific strategies for resource conservation of *O. japonicus* based on its genetic diversity and population structure.

**Supplementary Information:**

The online version contains supplementary material available at 10.1186/s12870-023-04247-2.

## Background

Phylogeography, which is a discipline concerned with various relationships between gene genealogies, phylogenetics and geography [1, 2], is often applied to compare the genetic lineages and current geographical distribution patterns of species to explore the historical processes and provide useful information for sustainable cultivation of plants. The currently DNA molecular marker techniques used in the analysis of plant phylogeography mainly include chloroplast DNA (cpDNA), nuclear DNA (nDNA) and simple sequence repeat (SSR). Although nDNA and SSR have a faster evolution rate than cpDNA and are inherited biparentally, a phenomenon termed concerted evolution has made nDNA more suitable for studying phylogeography among species [3], while the disadvantage of SSR easily losing some gene information in the process of mutation limits its application in phylogeography [4]. For cpDNA, the traits of uniparental inheritance and haploid enable it to preserve the genetic traces of plants in the long history process to a large extent. Besides, a shorter mean coalescent time gives cpDNA a greater advantage in calculating the time of ancestral differentiation [5]. Therefore, it is appropriate to use phylogeographic approaches based on cpDNA to understand the demographic and historical processes of medicinal plants and to further propose suitable conservation and sustainable cultivation practices for medicinal plants.

*Ophiopogon japonicus* (L. f.) Ker-Gawl., an evergreen perennial herb in the Liliaceae family. is a typical domesticated medicinal plant whose cultivation history can trace back to Song Dynasty. The tuberous root of *O. japonicus* has been used for the treatment of coughs, sore throats, thirstiness, insomnia and constipation in traditional Chinese medicine (TCM) over 2000 years due to its functions in nourishing *Yin*, promoting the production of body fluid, moistening lungs and clearing away internal heat [6]. Zhe maidong (ZMD) and Chuan maidong (CMD) are two genuine cultivars of *O. japonicus* in China planted in Zhejiang and Sichuan respectively. Different geographical locations led to the different growing environment of the two cultivars [7]. Despite these external factors, CMD and ZMD have had stable mutation loci in cpDNA and nuclear genome [7, 8]. All these phenomena may cause different chemical constituents [9–11] and pharmacological activities [12–14] between ZMD and CMD, which would play pivotal roles in recognizing a superior clinical efficacy of ZMD over CMD. However, previous studies revealed that the genetic diversity of *O. japonicus* has decreased a lot which will affect the long-term survival and evolutionary potential of this species, and then affect the selection and breeding of high-quality medicinal herbs [15, 16]. Hence, further evaluation of the genetic diversity and location of the original site of cultivated *O. japonicus* are essential for designing strategies of protecting the genetic resources and breeding new varieties.

To provide practical information necessary for origin exploration and sustainable cultivation of *O. japonicus*, we used 4 chloroplast DNA regions (*atpB-rbcL*, *rpl16*, *psbA-trnH* and *rpl20-5’rps12*) to figure out the population genetics and phylogeography of wild and cultivated *O. japonicus*. More specific objectives are to (1) evaluate the genetic diversity of cultivated populations under artificial selection and wild populations, and determine which wild populations can be introduced for the improvement of cultivation; (2) clarify the genetic divergence between cultivated populations of *O. japonicus* from Sichuan province and Zhejiang province; and (3) disclose the geographical origins and ancestral populations of cultivated *O. japonicus*.

## Results

### Genetic diversity and differentiation of ***O. japonicus***

One cpDNA intron (*rpl16*) and three cpDNA spacers (*atpB-rbcL*, *psbA-trnH* and *rpl20-5’rps12*) were chosen to analyze the genetic variation in *O. japonicus* from different geographical locations after preliminary variation screening. Based on 266 individuals representing 23 populations of *O. japonicus*, the nucleotide sequence length was 698 bp, 523 bp, 479 and 708 bp for the *atpB-rbcL*, *rpl16*, *psbA-trnH* and *rpl20-5’rps12* regions, respectively. The combined cpDNA sequence length after multiple alignments was 2408 bp. In total, 107 polymorphic sites and 19 haplotypes were detected from 10 cultivated populations and 13 wild populations of *O. japonicus*. The variation rate of the combined cpDNA sequence was 4.44%, and the aligned sequence variations are summarized in Additional file 1.

**Fig. 1:**
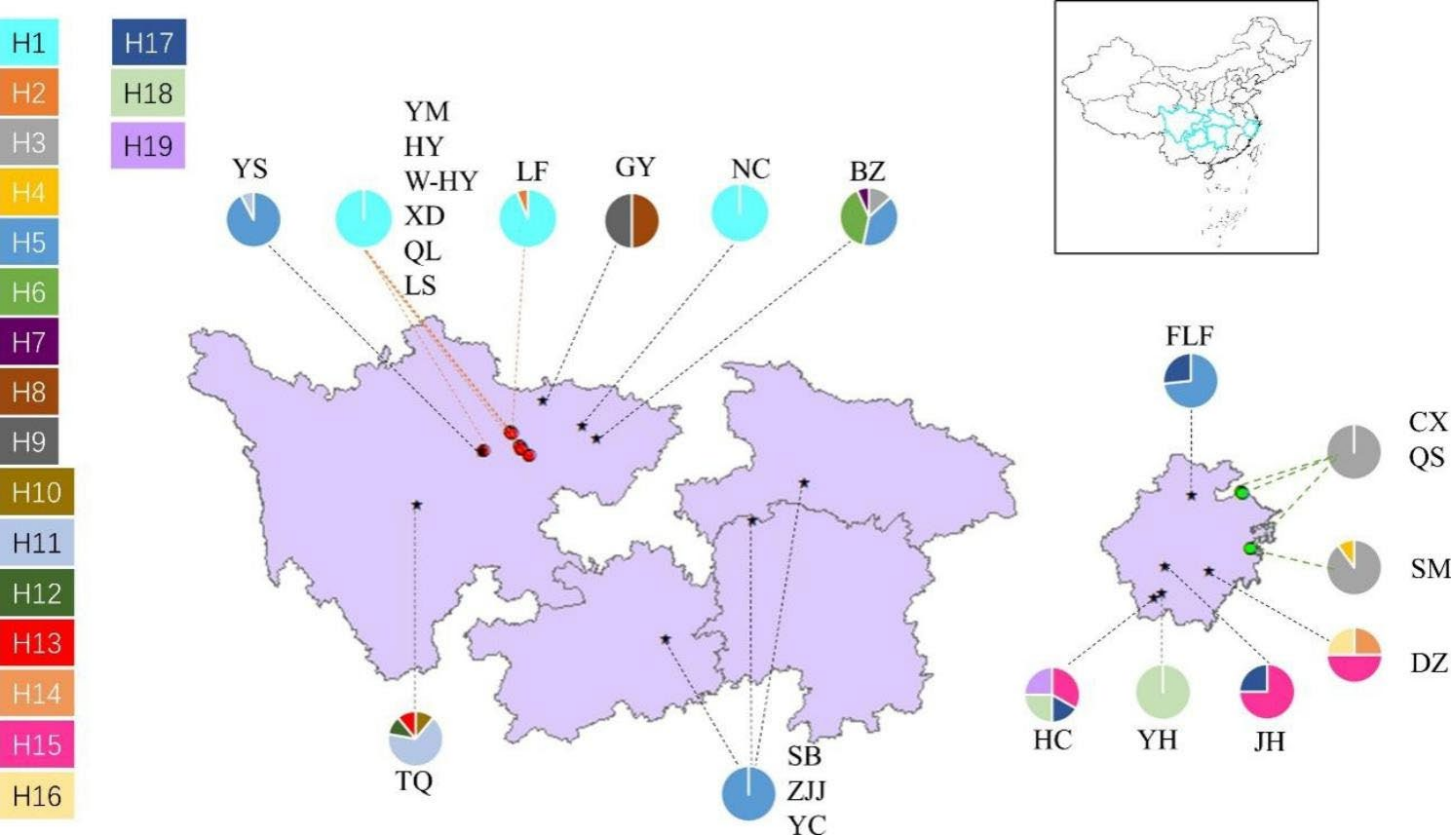
A geographic distribution of 19 cpDNA haplotypes (H1-H19) detected in *O. japonicus*

Genetic diversity of *O. japonicus* was estimated by the number of haplotypes (H), haplotype diversity (*H*_d_) and nucleotide diversity (*π*) in *O. japonicus* (Table 1). 19 haplotypes were obtained from 23 populations, including 4 haplotypes from cultivated *O. japonicus* and 15 haplotypes from wild *O. japonicus* (Fig. 1). The *H*_d_ and *π* at the species level of *O. japonicus* were 0.74500 and 0.00712 respectively. The *H*_d_ and *π* of CMD (0.01900; 0.00006) were lower than those of ZMD (0.06900; 0.00049) and wild populations (0.76200; 0.00905). Furthermore, the result of permutcpSSR showed that the genetic diversity of all 13 wild populations (*h*_T_ = 0.849, *h*_S_ = 0.341, *V*_T_ = 0.859, *V*_S_ = 0.204) was much higher than that of the two cultivars (*h*_T_ = 0.521, *h*_S_ = 0.030, *V*_T_ = 0.842, *V*_S_ = 0.067).

According to the AMOVA analysis (Table 2), genetic differentiation of cultivated *O. japonicus* occurred mainly between the two cultivars (95.51% among populations in the nonhierarchical AMOVA analysis of cultivated, and 98.37% among groups in the hierarchical AMOVA analysis of CMD vs. ZMD), which is different from wild *O. japonicus* whose variation mainly occurred among populations (70.09%). Comparison of different groups showed huge genetic variations between CMD and ZMD (98.37%), which is consistent with the low gene flow between the two cultivars (*N*_m_ =0.04). The results suggest that there had been a high genetic differentiation between CMD and ZMD. What’s more, the percentage of genetic variation between CMD and wild group (40.30%) was much higher than that between ZMD and wild group (8.64%), because the gene flow between ZMD and wild group (*N*_m_ = 1.490) can resist genetic differentiation caused by genetic drift among populations between ZMD and wild groups (*F*_ST_ = 0.72934), while the gene flow between CMD and wild group (*N*_m_ = 0.830) was insufficient to resist the genetic differentiation caused by migration or genetic drift between CMD and wild group (*F*_ST_ = 0.82566).

**Table 1:** Genetic diversity analysis based on cpDNA of *O. japonicus*

Population code	H	*H*_d_	*π*	Tajima *D*	Fu’s *F*S
Cultivated in Sichuan (CMD)					
YM	1	0	0	0	0
HY	1	0	0	0	0
W-HY	1	0	0	0	0
XD	1	0	0	0	0
QL	1	0	0	0	0
LF	2	0.125	0.00036	-2.06208	2.38195
				**	
LS	1	0	0	0	0
Species level	2	0.019	0.00125	-2.09486	-0.02002
				***	
Cultivated in Zhejiang (ZMD)					
FM	1	0	0	0	0
QS	1	0	0	0	0
SM	2	0.2	0.00141	-2.01902	5.88323
				***	
Species level	2	0.069	0.01096	-2.51175	3.84807
				***	
Wild					
YS	2	0.154	0.00211	-2.30289	8.81879
				***	
GY	2	0.566	0.00046	1.84427	2.42917
TQ	4	0.533	0.00232	-0.33388	3.34381
BZ	4	0.705	0.00488	0.52501	9.40284
NC	1	0	0	0	0
FLF	2	0.419	0.004	0.64702	12.53529
JH	2	0.429	0.00036	0.41421	1.65331
DZ	3	0.833	0.00568	-0.74309	3.15685
HC	4	0.803	0.01341	2.7618	14.2524
YH	1	0	0	0	0
SB	1	0	0	0	0
ZJJ	1	0	0	0	0
YC	1	0	0	0	0
Species level	17	0.762	0.20378	0.60829	20.39273

Note: H, the number of haplotypes; *H*_d_, haplotype diversity; *π*, nucleotide diversity. *P < 0.05, ** *P* < 0.01, *** *P* < 0.001

**Table 2:** Analysis of molecular variance (AMOVA) based on the cpDNA sequences

Source of variation	d.f.	Sum of squares	Variance components	Percentage of variation	Fixatio indices	*P*-value
Species						
Among populations	23	1895.165	7.10101 Va	80.87	*F*_ST_ = 0.78097	0
Within populations	250	419.988	1.67995 Vb	19.13		
Wild						
Among populations	12	1016.788	8.09993 Va	70.09	*F*_ST_ = 0.70087	0
Within populations	119	411.378	3.45696 Vb	29.91		
Cultivated						
Among populations	9	452.518	3.75356 Va	95.51	*F*_ST_ = 0.95514	0
Within populations	124	21.863	0.17631 Vb	4.49		
Cultivated vs. Wild						
Among groups	1	369.238	2.19366 Va	22.09	*F*_SC_ = 0.76952	0
Among populations within groups	21	1469.307	5.95263 Vb	59.95	*F*_ST_ = 0.82044	0
Within populations	243	433.241	1.78288 Vc	17.96	*F*_CT_ = 0.22093	0.0081
CMD vs. ZMD						
Among groups	1	451.033	9.92018 Va	98.25	*F*_SC_ = 0.00383	1
Among populations within groups	8	1.485	0.00068 Vb	0.01	*F*_ST_ = 0.98254	0
Within populations	124	21.863	0.17631 Vc	1.75	*F*_CT_ = 0.98247	0.00502
CMD vs. Wild						
Among groups	1	585.659	4.45178 Va	40.3	*F*_SC_ = 0.70800	0
Among populations within groups	18	1017.159	4.66986 Vb	42.27	*F*_ST_ = 0.82566	0
Within populations	217	417.94	1.92599 Vc	17.43	*F*_CT_ = 0.40296	0
ZMD vs. Wild						
Among groups	1	117.718	0.93887 Va	8.64	*F*_SC_ = 0.70376	0
Among populations within groups	14	1017.902	6.99048 Vb	64.3	*F*_ST_ = 0.72934	0
Within populations	145	426.678	2.94261 Vc	27.07	*F*_CT_ = 0.08636	0.13675

Note: *F*_ST_: Genetic differentiation among populations; *F*_SC_: genetic differentiation among population within groups; *F*_CT_: Genetic differentiation between groups

### Population structure and phylogeographical analysis

**Fig. 2:**
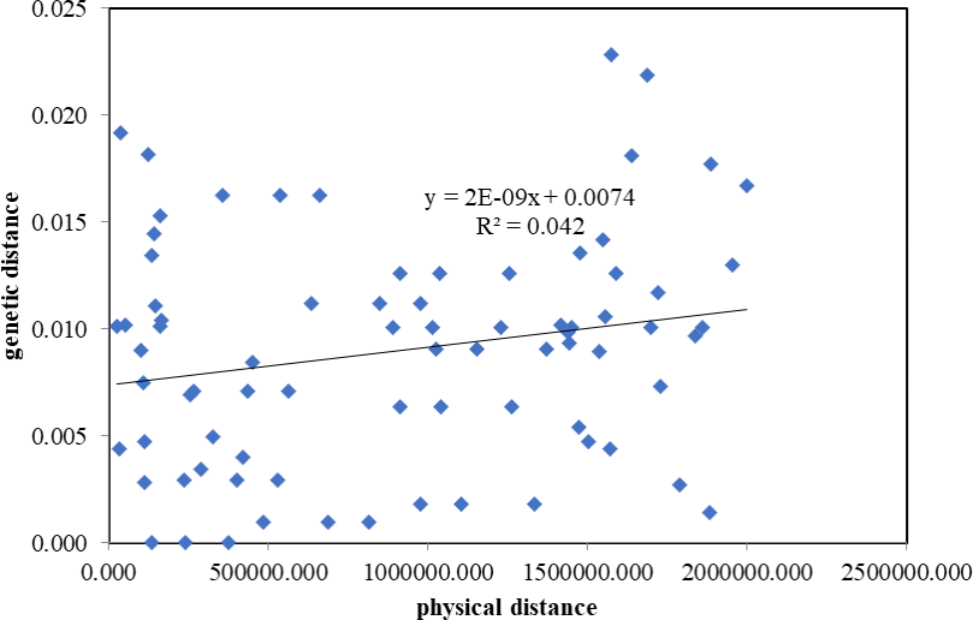
Mantel test of relationship between genetic distance and geographical distance among populations of *O. japonicus*

A permutation test showed that *N*_ST_ was significantly higher than *G*_ST_ (*N*_ST_/*G*_ST_ = 0.920 /0.753, *P* < 0.01), indicating that geographically close haplotypes were distributed in the same or contiguous population. And the genetic differentiation among population increased with the increase of geographical distance, indicating that there was a significant phylogeographic structure in *O. japonicus* cpDNA haplotypes. Mantel test, in which only wild populations were included, also supported the result that the genetic distances between *O. japonicus* populations were significantly and positively correlated with their geographical distances (*P* = 0.05, R^2^ = 0.111). With the increase of geographical distance, the genetic distance of *O. japonicus* also showed a trend of increase (Fig. 2). Combined with the low gene flow among populations of *O. japonicus* (*N*_m_ = 0.17), this species showed a pattern of restricted gene flow with isolated by distance.

**Fig. 3:**
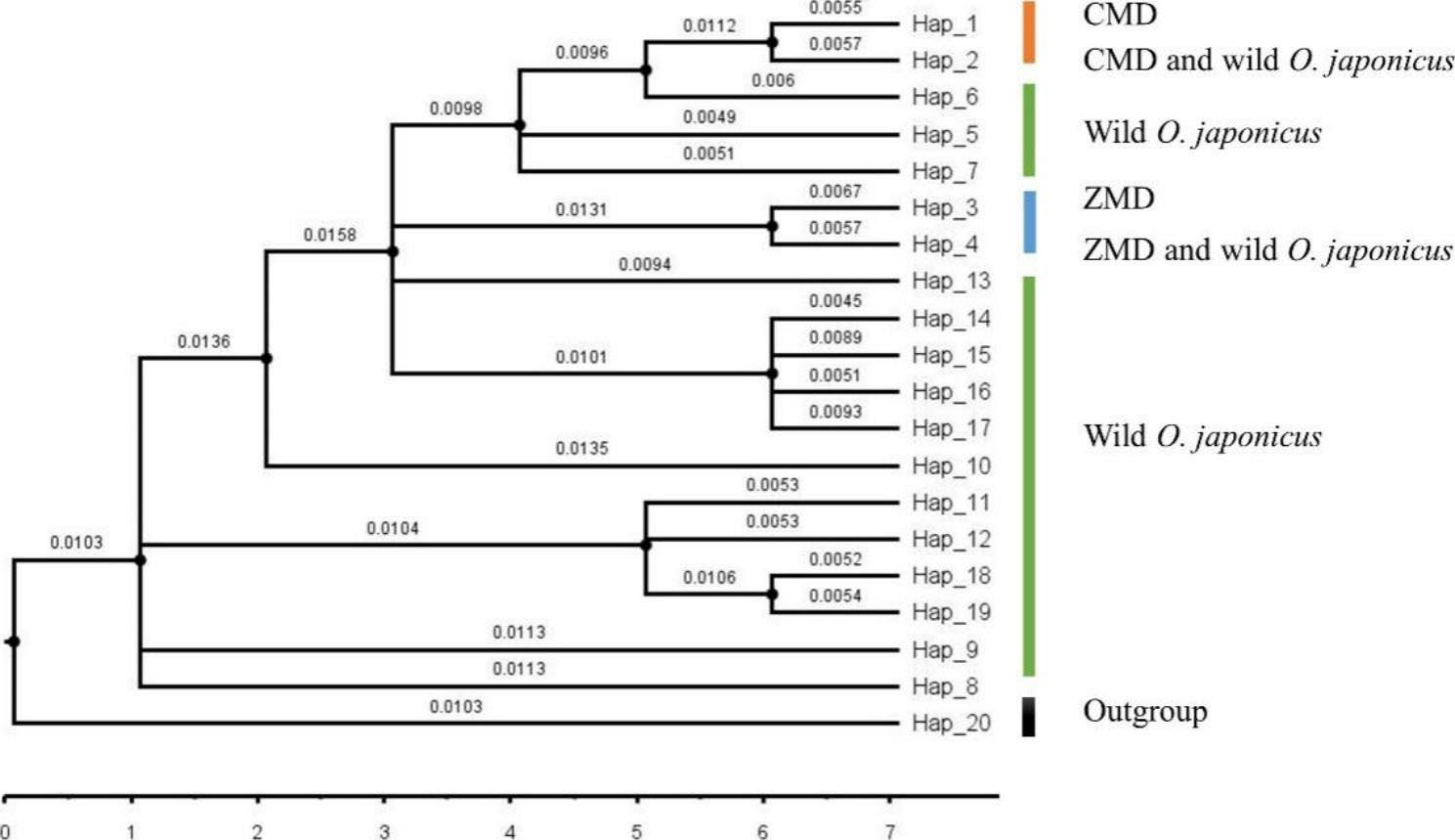
Maximum-likelihood (ML) phylogenetic tree: the number showed on nodes stands for the bootstrap value on 1000 replicates; *O. amblyphyllus* Wang et Dai is set as an outgroup

The phylogenetic relationships between the 19 cpDNA haplotypes were assessed under Bayesian inferences drawn using *O. amblyphyllus* Wang et Dai (H20) as an outgroup. The result showed that the phylogenetic tree of the 19 cpDNA haplotypes had a comb-like structure (Fig. 3). Haplotypes (H1, H2, H3 and H4) from cultivated populations occurred in different clades, but still grouped together compared with the outgroup. As a result, we could not distinguish CMD from ZMD based on the 4 cpDNA sequences.

**Fig. 4:**
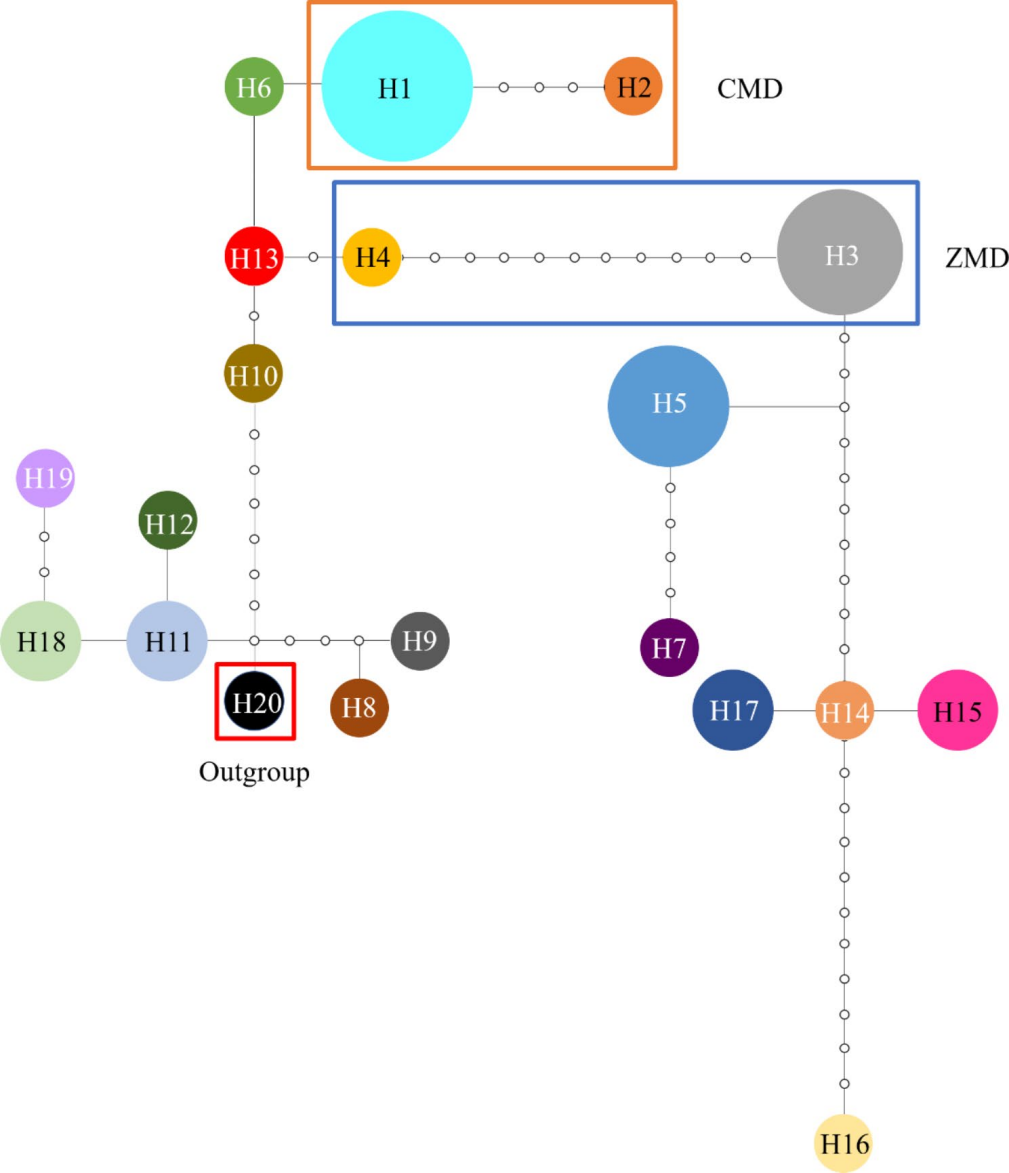
Minimum spanning network of 19 cpDNA haplotypes in *O. japonicus*. The network was rooted at the *O. amblyphyllus* Wang et Dai. The circle size is proportional to the number of samples per haplotype. Hollow dots indicate the number of mutational steps

Furthermore, the rooted TCS network of cpDNA haplotypes revealed the relationship of the interior (ancestral) and the tip (derived) haplotypes (Fig. 4). H13, the haplotype only found in the TQ population, was inferred as the ancestral haplotype because it lies at the center of TCS network and is connected with other derivative haplotypes. 4 cpDNA haplotypes (H1, H2, H3 and H4) were found within the cultivated populations and all of them came from H13. Among them, H1 was the most frequent and widely distributed haplotype and shared between CMD and wild *O. japonicus*; H3 was shared between ZMD and wild *O. japonicus*; H2 was shared only by CMD; and H4 was shared only by ZMD. The other 15 haplotypes were found only in wild populations. The haplotype distribution also confirmed the low genetic diversity of cultivated *O. japonicus*.

### Demographic history

**Fig. 5:**
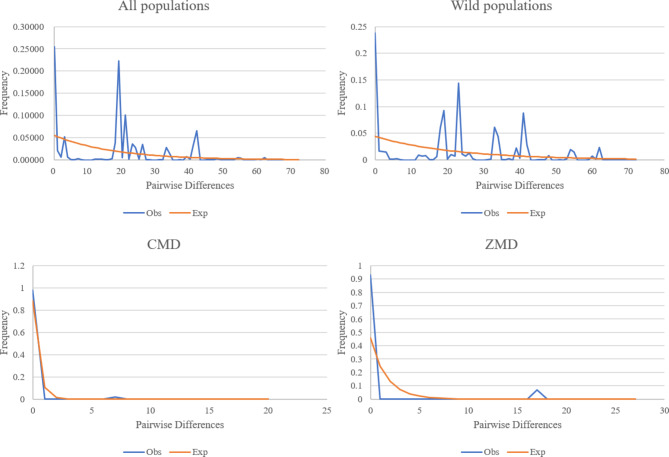
Mismatch distribution for cpDNA sequence data in *O. japonicus*. The red line represents the expected values and blue line represents the observed values

The mismatch distribution for *O. japonicus* was clearly multimodal and inconsistent with the bell-shaped curve expected for an expanding population at species level (SSD = 0.10627, *P* = 0.000 < 0.05), suggesting that *O. japonicus* did not undergo a recent expansion in the face of favorable climatic and environmental conditions (Fig. 5). Neither did the wild population of *O. japonicus* expand recently (SSD = 0.08589, *P* = 0.000 < 0.05). However, CMD whose SSD-value was 0.00028 (*P* = 0.058 > 0.05) and ZMD whose SSD-value was 0.00401 (*P* = 0.160 > 0.05) had a relatively smooth unimodal distribution, suggesting that CMD and ZMD could be assumed as an expansion model.

At the species level, both Tajima’s *D* (-0.03985, *P* = 0.799) and Fu’s *F*s (20.36184, *P* = 0.926) showed no significant difference from zero, indicating *O. japonicus* had not passed through a recent demographic expansion. Neither did the wild populations undergo expansion recently (Tajima’s *D* = 0.60829, *P* = 0.732; Fu’s *F*s = 20.39273, *P* = 1.000). However, the value of Tajima’s *D* and Fu’s *F*s (Table 1) implied that demographic expansion existed in certain populations from CMD and ZMD, which is consistent with the result of mismatch distribution.

### Historical biogeography

**Fig. 6:**
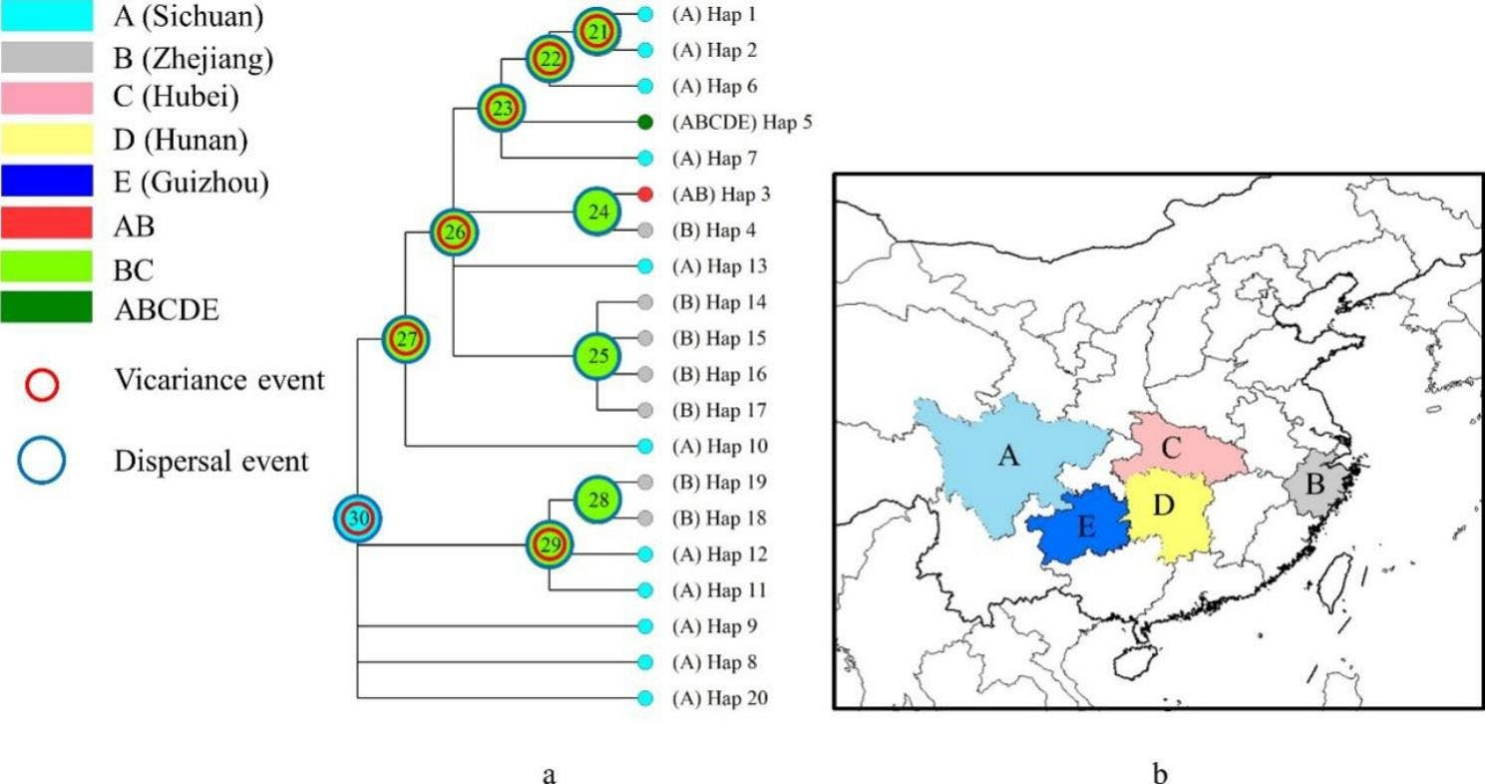
Historical biogeography of *O. japonicus.* (a) The ancestral area reconstruction results based on RASP of *O. japonicus*. The pie charts on nodes display the relative probabilities of possible ancestral ranges. (b) The map showing five geographical regions in colors as defined in RASP analysis.

The results of S-DIVA ancestral area reconstruction based on RASP suggested that *O. japonicus* may originate in Sichuan (A) with subsequent dispersal and vicariance events between the ancestral and areas of present distribution (Fig. 6). These facts support that Sichuan may be an ancestral area of *O. japonicus*.

### Species distribution modeling

**Fig. 7:**
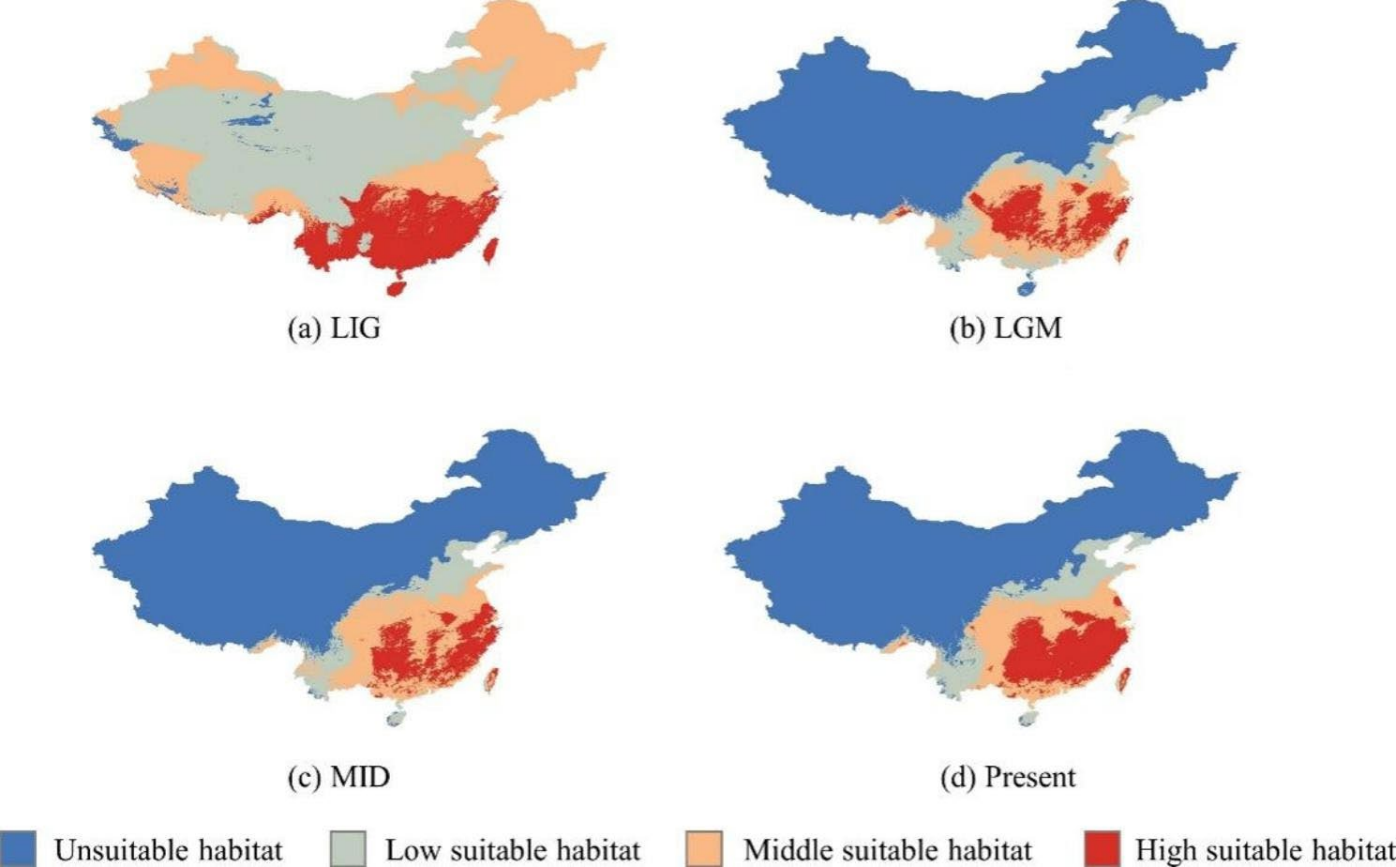
Simulation of the distribution area of *O. japonicus* in different climatic conditions in China

Based on the geographical distribution of *O. japonicus*, MaxEnt was used to simulate the potential distribution of *O. japonicus* during LIG, LGM, MID and present (Fig. 7). The AUC score, which represents the average of 10 runs, was higher than 0.8 for the four models built, which supports their predictive power [17].

The simulation results of species distribution showed that *O. japonicus* distributed in most areas of China during the LIG period (Fig. 7-A). After the LGM period, the distribution ranges of the species had a slight expansion or recolonization in the geographical range (Fig. 7-C). While the distribution range of *O. japonicus* at present is similar to that of MID (Fig. 7-D), indicating that the migration and expansion of *O. japonicus* at MID tend to be basically stable.

## Discussion

### Genetic diversity and differentiation

For many cultivated crops, artificial selection, founder effect and genetic bottleneck may consequently reduce their genetic diversity [18–20], as well as their capacity for long-term survival and evolution in dynamic environments. Due to the comparatively brief domestication period and the exchange of genes with wild populations during extensive cultivation, the genetic diversity of medicinal plants has declined in a much milder manner as compared with the crops. In our fieldwork, we investigated 13 sites of wild *O. japonicus*, 7 CMD cultivation bases in the villages along Fujiang River in Sichuan province and 3 ZMD cultivation bases, 2 of which are situated in the traditional genuine producing area Cixi and the other is located in new producing area Santai. The present study demonstrated that cultivated *O. japonicus* had undergone a striking decrease in genetic diversity (*h*_T_ = 0.521, *h*_S_ = 0.030, *V*_T_ = 0.842, *V*_S_ = 0.067) compared with the wild populations (*h*_T_ = 0.849, *h*_S_ = 0.341, *V*_T_ = 0.859, *V*_S_ = 0.204) after domestication for several hundred years. Of the 19 cpDNA haplotypes in the species, only 4 haplotypes were detected in cultivated populations. The domestication result of decreased genetic diversity is similar to that of *Scrophularia ningpoensis* [21] and *Angelica dahurica* [22]. These dramatic changes in the genetic diversity of *O. japonicus* during such a short domestication history are most likely caused by founder effect and the clonal mode of reproduction. Firstly, the founder effect is that when a population is established and developed by a small number of individuals, the genetic information carried by these few individuals does not completely reflect the genetic information of their source population, resulting in a low genetic diversity of the new population. The loss of genetic diversity for medical plants like *Allium mongolicum* [23], *Leonurus cardiaca* [24] and *Lycium barbarum* [25] is more or less related to the founder effect. In this study, we only gathered 2 haplotypes of cultivated *O. japonicus* from the haplotypes of wild populations due to the founder effect, meaning that the founders contained only a few samples of the wild populations with certain genetic information. Secondly, although the domestication history of *O. japonicus* was shorter than other medical plants like *Coix lacryma-jobi* L. and *Carthamus tinctorius* L. [26], the genetic diversity of cultivated *O. japonicus* was dramatically decreased because wild *O. japonicus* could expand via fruits while cultivated *O. japonicus* was influenced by asexual reproduction and artificial selection. In addition, previous studies have demonstrated that genetic bottlenecks caused by domestication in annual and perennial fruit crops, suggesting that perennial crops can preserve a higher percentage of the genetic variation present in their wild progenitors than annual crops [27]. And juvenile phase length is a principal difference between domesticated annual and domesticated perennial fruit crops. In this study, the cultivation time before harvest was 2 or 3 years for ZMD and 1 year for CMD. ZMD (*H*_d_ = 0.06900; *π* = 0.00049) showed a higher genetic diversity than CMD (*H*_d_ = 0.06900; *π* = 0.00049). And the gene flow of ZMD-wild (*N*_m_ = 1.490) was higher than that of CMD-wild (*N*_m_ = 0.830), implying that the relatively lengthy juvenile phases coupled with ongoing cultivar–wild gene flow due to the change of habitat may contribute to the milder genetic bottlenecks in perennial ZMD than annual CMD, resulting in relatively high genetic diversity in ZMD.

In addition to reducing the genetic diversity of medical plants, domestication also affects the structural patterns of genetic variations [28]. For instance, notable genetic divergence was found between wild and cultivated *Angelica dahurica* and between its two cultivars (*F*ct = 0.148, *P* = 0.000) [22]. Our result of AMOVA analysis indicated that 21.8% of the total genetic variation occurred between cultivated and wild *O. japonicus*, while 98.37% of the total genetic variation occurred between CMD and ZMD (Table 2). Further analysis revealed that the genetic differentiation between ZMD and wild *O. japonicus* (*F*_ST_ = 0.72934) was lower than that between CMD and wild *O. japonicus* (*F*_ST_ = 0.82566). The results of mismatch distribution and neutrality test indicated that both ZMD and CMD had undergone a recent population expansion. Frequent extinctions and recolonizations of local populations can also be an important source of gene flow[29]. It has been reported that ZMD production areas have experienced significant changes in recent years [30]. Due to urbanization and regional economic reasons, most farmers in the traditional production area of ZMD (Hangzhou) no longer plant ZMD. Sanmen County, located in Taizhou City, Zhejiang province, has gradually became a new producing region of ZMD. As a result, it is probable that one of the factors contributing to the higher gene flow between ZMD and wild populations (*N*_m_ = 1.490) than between CMD and wild populations (*N*_m_ = 0.830) is the shrinkage of traditional production regions and the creation of new production areas. In a group of completely isolated populations, genetic drift tends to fix different alleles in different local populations. Genetic drift is the unpredictable change in gene frequency due to finite population size. Gene flow between any populations will prevent complete fixation, but gene flow must exceed a certain level (*N*_m_ > 1) to prevent substantial genetic differentiation due to genetic drift [29]. This also clarifies why the gene flow between ZMD and wild populations (*N*_m_ = 1.490) is able to withstand the genetic differentiation caused by genetic drift while the gene flow between CMD and wild populations (*N*_m_ = 0.830) is unable.

As for the genetic differentiation between CMD and ZMD, on the one hand, we believe that it is the result of artificial selection and the isolation by distance. Our surveys conducted in the cultivated regions indicated that seedlings of CMD are prostrate CMKY-2, whereas the seedlings of ZMD are mainly erect *O. japonicus* with a small number of prostrate *O. japonicus* [31]. This combination of artificial selection and clonal propagation of *O. japonicus* rapidly fixes the alleles carried by selected individuals, which means that one or two haplotypes are fixed in each cultivar population, leading to large genetic differences between ZMD and CMD. In addition, the herbal textual studies and the description of the local planters exhibited that there was no record about the introduction of one cultivar of *O. japonicus* into another cultivar’s growing region, which may have prevented gene flow and then leading to the genetic differentiation between CMD and ZMD. Finally, population differentiation is also influenced by geographical distance. Isolation by distance is a model of the population structure in which genetic differences between populations increase with the geographic scale [32]. In other words, populations adjacent to each other share more similarities than those growing far apart, because they are linked by larger gene exchange, unless some impassable isolation breaks this mechanism and reduces the communication between some populations and their neighbors. Our results showed that a greater *N*_ST_ (0.920) than *G*_ST_ (0.753), indicating the presence of the phylogeographical structure of *O. japonicus*. Combined with the result of mantel test, it can be inferred that the restricted gene flow with isolated by distance contribute to the population structure of *O. japonicus* [33]. Geographical isolation can contribute to the lack of gene flow among populations leading to the genetic differentiation between two cultivars.

### Origin of cultivated ***O. japonicus***

Designing strategies for the protection of genetic resources and the breeding of new varieties can be assisted by identifying the original site of domestication and evaluating the evolution history of wild-domesticated species [34]. Before Tang Dynasty, *O. japonicus* was believed to come from the wild species and be mainly distributed in Henan, Jiangsu, Zhejiang and Anhui provinces. ZMD and CMD cultivation can trace back to Song Dynasty and Ming Dynasty respectively [35]. As the first study exploring the origin of *O. japonicus*, we explored the ancestral distribution of *O. japonicus* and its historical changes based on 4 chloroplast DNA data. ML phylogenetic tree in this study showed that ZMD and CMD were grouped as a cluster as compared with the outgroup (Fig. 3). Further studies found ZMD and some individuals in the wild population BZ located in Sichuan were grouped as a cluster, while CMD and the wild population NC located in Sichuan were grouped as another cluster. These results suggest that the cultivated *O. japonicus* is more closely linked to wild populations in Sichuan than the wild *O. japonicus* in Zhejiang, which is similar to the study on the genetic diversity of cultivated *O. japonicus* based on ISSR markers [15]. Therefore, the wild populations NC and BZ located in Sichuan province may have played an important role in the origin of cultivated *O. japonicus*.

The TCS network claimed that 2 haplotypes (H1 and H2) found in CMD and 2 haplotypes (H2 and H4) found in ZMD were derived from H6 and H13 respectively. The 2 haplotypes (H6 and H13) both found only in the wild population BZ and TQ located in Sichuan province. All of the aforementioned haplotypes were derived from H13, which lied at the center of the haplotype network and was only found in the wild population TQ located in Sichuan. It may be the ancestor of the two cultivars in this study. Furthermore, as the most frequent and widely distributed haplotype, H1 was found not only in CMD but also in the wild population NC, while H3, which occurred in most ZMD, was also found in the wild population BZ, suggesting that the wild populations in Sichuan contributed more to the origin of *O. japonicus*.

The result of historical biogeography and the model of species distribution indicated that *O. japonicus* distributed in most areas of China during LIG period (Fig. 7-A). But the cold climate deprived the previously hospitable environment of this species, leading to a large-scale migration. *O. japonicus* migrated to the east of Hengduan Mountains and south of Qinling Mountains (Fig. 7-B), suggesting that these mountains may have been the refuge of this species during LGM. The ancestor of *O. japonicus* was located in Sichuan at that time, and then experienced multiple vicariance and dispersal processes between Sichuan and Zhejiang due to the emergence of suitable climatic conditions after LGM. This may explain why haplotypes H5 appeared in different populations located in Sichuan, Zhejiang, Hubei, Hunan, Guizhou and other provinces. In addition, the glacial refugium hypothesis (GRH) proposes that glaciers promoted differentiation and generation of intraspecific diversity by isolating populations in ice-free refugia [32], which was also supported by the results of haplotype diversity, geographical analysis and historical biogeography.

In summary, *O. japonicus* was widely distributed in China during the LIG period. With the passage of time, the cold climate caused *O. japonicus* to migrate to the east of Hengduan Mountains and south of Qinling Mountains during the LGM period. The present distribution was formed after several vicariance-dispersal events on the basis. More importantly, wild populations similar to the extant TQ located in Sichuan province may be the ancestor of cultivated *O. japonicus*.

### Sustainable cultivation of ***O. japonicus***

Wild populations have been considered as critical resources for breeding medical plants because their genetic information are used infrequently and generally exhibit no reproductively isolation from cultivated populations [36]. Our study compared cultivated and wild populations of *O. japonicus* indicated that cultivated *O. japonicus* had much lower genetic diversity than wild populations (Fig. 1; Table 1). The large-scale cultivation of *O. japonicus* has affected the genetic diversity of wild populations. For example, the wild population NC showed a single and identical haplotypes (H1) to CMD, which may reduce the plasticity of cultivated *O. japonicus* to respond to changes in climate, pathogen populations, agricultural practices, or quality requirements. Hence, it is necessary to improve the genetic diversity of cultivated *O. japonicus* through sexual reproduction by using wild resources.

Accumulative evidence indicates that a proper assessment of genetic resources of medicinal plants can provide useful information for the development of conservation plans to protect genetic diversity. For example, protection of medicinal plants like *Angelica dahurica* [22], *Aspidopterys obcordata* var. *obcordate* [37] and *Vitex rotundifolia* [38] were mostly based on the conservation of genetic diversity. Our study exhibited that wild populations DZ, HC and BZ had more cpDNA haplotypes and higher genetic diversity (*H*_d_ = 0.83300, *π* = 0.00568; *H*_d_ = 0.80300, *π* = 0.01341; *H*_d_ = 0.70500, *π* = 0.00488) than other populations (Fig. 1; Table 1). In addition, populations located in glacial refugia should be considered the priority for conservation because they not only preserve the genetic differentiation of species during the period of climate upheaval, but also serve as the starting point for post-glacial re-dispersal of species which can continue to maintain the continuity of genetic diversity [39–41]. The ENM results suggested that the east of Hengduan Mountains and south of Qinling Mountains were the glacial refugia of *O. japonicus* during LIG. RASP analysis indicated that the ancestor of *O. japonicus* was in Sichuan. These results demonstrated that Sichuan should be the starting point for post-glacial re-dispersal of *O. japonicus*. In summary, populations DZ, HC and BZ with high genetic diversity and populations TQ and BZ located in Sichuan with high genetic diversity should serve as key objects for biodiversity protection of *O. japonicus* and preferentially selected as the gene pool for improving the genotypes of cultivated *O. japonicus*.

## Conclusion

In this study, we accessed the level of genetic structure of *O. japonicus* from 23 sampling sites by using 4 maternally inherited cpDNA primers and investigated the origin of *O. japonicus* for the first time. The result showed that it was the anthropogenic influence that led to the low genetic diversity and a high degree of genetic differentiation of *O. japonicus* from two producing areas as well as the high degree of genetic differentiation between the cultivated and wild populations. In terms of the origin, Sichuan may be one of the origins of *O. japonicus*, and the wild populations TQ in Sichuan area were recognized as the ancestor of cultivated *O. japonicus*. In addition, we postulate that the populations containing high genetic diversity (DZ, HC and BZ) and populations distributing in glacial refugia with high genetic diversity (TQ and BZ) should be protected for the conservation of genetic resource and improvement of genetic diversity for *O. japonicus*. Our findings may help better understand the influence of domestication on genetic diversity of medicinal plants and provide genetic insights into conservation and sustainable cultivation of *O. japonicus*.

## Materials and methods

### Plant material and DNA extraction

**Fig. 8:**
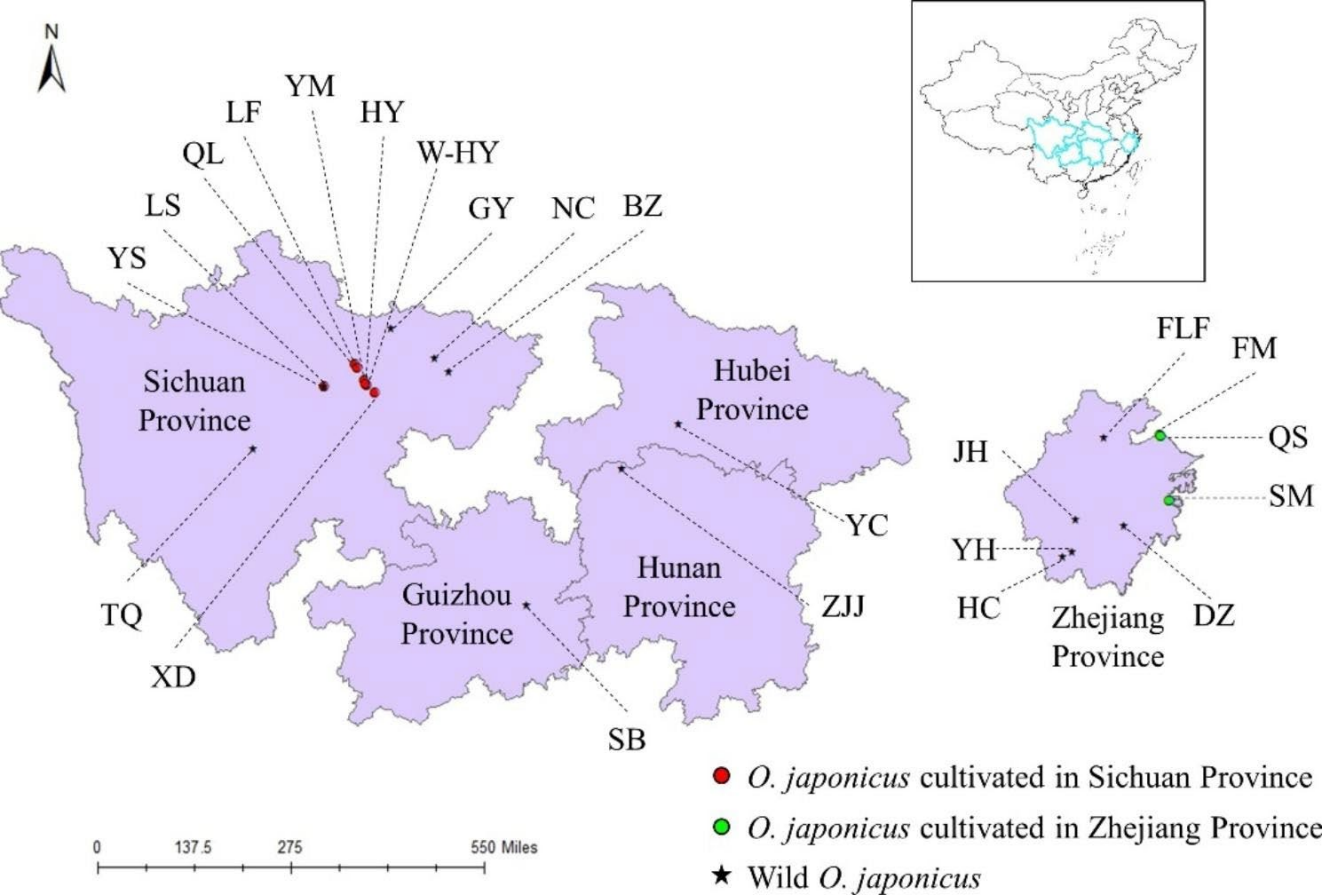
Sample locations of *O. japonicus* used in this study

A total of 266 individual *O. japonicus* plant samples were obtained from 23 sites of different distribution areas of China for cpDNA analysis (Fig. 8; Table 3). The plants from which leaves were collected were apart from each other for more than 20 m to avoid overlapping clones (genotypes). The leaf samples were dried in silica gel and then stored in a freezer (− 20℃) for use. A sample of *O. amblyphyllus* Wang et Dai from Sichuan was also collected as the outgroup. Genomic DNA was extracted using the Plant Genomic DNA kit (Tiangen Biotech, Beijing, China). For the wild and cultivated plants we used in this study, we obtained relevant permission to collect. All voucher specimens were morphologically identified by Professor Luping Qin from the College of Pharmaceutical Sciences, Zhejiang Chinese Medical University (ZCMU) and deposited at the herbarium of ZCMU (voucher ID numbers: 510000190328LY001-510000190328LY013; 330000190630LY001-330000190630LY009; 520000200602LY001; 430000200527LY001; 420000200530LY001). Our field study and experimental research complied with local legislation, national and international guidelines.

**Table 3:** Details of sample locations and sizes

Population code	Locality	Longitude (E)	Latitude (N)	Altitude	Sample size
				(m)	
Cultivated in Sichuan (CMD)					
YM	Tianping, Yongming, SiChuan, China	104°51′58.40”	31°21′38.04”	417	15
HY	Xinglou, Huayuan, SiChuan, China	104°55′05.10”	31°16′08.28”	411	15
W-HY	Qinshan, Huayuan, SiChuan, China	104°53′36.44”	31°17′19.59”	417	15
XD	Maji,Xinde, SiChuan, China	105°05′19.91”	31°07′50.30”	367	15
QL	Wenfeng, Qinglian, SiChuan, China	104°40′40.27”	31°40′20.50”	496	15
LF	Shunjiang, Longfeng, SiChuan, China	104°42′40.61”	31°37′08.97”	480	16
LS	Luocheng, Luoshui, SiChuan, China	104°02′36.99”	31°14′58.92”	675	14
Cultivated in Zhejiang (ZMD)					
FM	Fumin, Andong, ZheJiang, China	121°13’48.21”	30°18′00.94”	3	10
QS	Fujia road, Chongshou, ZheJiang, China	121°15’18.54”	30°15′49.99”	4	10
SM	Nanlin, Hengdu ZheJiang, China	121°26’33.72”	29°00′33.34”	60	10
Wild					
YS	Luocheng, Luoshui, SiChuan, China	104°01′45.66”	31°15′10.62”	817	13
GY	Yueba,Baichao, SiChuan, China	105°25′40.45”	32°22′58.33”	1470	10
TQ	Hongling mountain, Labahe, SiChuan, China	102°34′17.99”	30°02′27.81”	1663	10
BZ	Fangdou mountain, Qunle, SiChuan, China	106°36′28.89”	31°31′57.93”	585	15
NC	Liujiawan, Fengzhan, SiChuan, China	106°19′00.48”	31°48′48.82”	456	11
FLF	Feilai peak, Xihu, ZheJiang, China	120°06’09.84”	30°14’17.92”	90	15
JH	Daxikou, Xilian, ZheJiang, China	120°22’35.34”	28°53’47.66”	374	8
DZ	Danzhu, Danzhu, ZheJiang, China	120°31’02.29”	28°31’13.41”	402	4
HC	Huang, Longnan, ZheJiang, China	119°14’38.54”	27°55’00.70”	1430	12
YH	Meijiujian, Chongtou, ZheJiang, China	119°27’11.09”	28°01’05.23”	1306	2
SB	Wengtang, Liutang, Guizhou, China	108°12′27.39”	26°58′43.27”	762	13
ZJJ	Xiangshuijian, Sifangxi, Hunan, China	110°10′30.07”	29°39′16.85”	446	7
YC	Mawang mountain, Nanqiao, Hubei, China	111°20′42.22”	30°31′18.84”	204	12
Outgroup					
DY	Hongling mountain, Labahe, SiChuan, China	102°34′07.26”	30°02′56.08”	1787	1

### cpDNA sequencing

Polymerase chain reaction (PCR) was performed in an Veriti96 thermocycler (Thermofisher). The primers unsed in this study are *atpB-rbcL* (F: GTG GAA ACC CCG GGA CGA GAA GTA GT; R: ACT TGC TTT AGT TTC TGT TTG TGG TGA), *rpl16* (F: GAT GGC GGA ATG AAC CAA GA; R: CGT ACC CAT ATT TTT CCA CCA CGA C), *psbA-trnH* (F: ACT GCC TTG ATC CAC TTG GC; R: CGA AGC TCC ATC TAC AAA TGG) and *rpl20-5’rps12* (F: TTT GTT CTA CGT CTC CGA GC; R: GTC GAG GAA CAT GTA CTA GG). The reaction mixture (50 µL) contained 2 µL of total DNA, 1 µL of each primer, 21 µL of ddH_2_O, and 25 µL of 2× Trans Taq High Fidelity (HiFi) PCR SuperMix (Transgen Biotech, Beijing, China). Double-stranded DNA was amplified after 4-min incubation at 94℃; followed by 30 cycles at 94 ℃ for 50 s, 54℃ for 50 s, and 72 ℃ for 90 s; with a final extension at 72 ℃ for 7 min. PCR products were identified on 1.0% agarose gels, visualized with ultraviolet light and photographed by using DNA Marker DL2000 (Takara Bio Inc.). The PCR products with a single band were cleaned by a EasyPure PCR Purification Kit (TransGen Biotech. Beijing, China) for direct sequencing. Sequencing was conducted from both ends.

### Phylogenetic analyses

Geneious Pro V4.8.3 [42] was used to trim, assemble and align the DNA sequences. Bayesian phylogenetic analyses was performed on MrBayes 3.2.4 [43] to construct the phylogenetic tree based on the combined cpDNA data. The sequence evolution model T92 + G was evaluated by the Akaike Information Criterion (AIC) in Modeltest 3.7.0 [44]. Two independent analyses with four simultaneous Monte Carlo Markov Chains (MCMC) analyses were run for 10 million generations, with sampling done every 100 generations. The first 50,000 sampled trees were discarded as burn-in samples. A statistical parsimony haplotype network estimated the genetic relationships among haplotypes was built using TCS 1.21 [45].

### Population genetic diversity and genetic differentiation

The number of haplotypes (H), haplotype diversity (*H*_d_) and nucleotide diversity (*π*) of *O. japonicus* were computed by DnaSP v5.10 [46]. The program PermutCpSSR_1.2.1 [47] was used to calculate the average within-population gene diversity (*h*_S_), total gene diversity (*h*_T_) and the level of population differentiation at the species level (*G*_ST_), as well as the geographical total haplotype diversity (*V*_T_), geographical average haplotype diversity (*V*_S_) and an estimate of population subdivisions for phylogenetically ordered alleles (*N*_ST_) obtained by taking into account the similarities between haplotypes. *G*_ST_ and *N*_ST_ were used to assess the geographical structure that affected population differentiation. To evaluate the existence of correlation between the geographic and genetic distances, Mantel test was performed using GenAlEx v.6.502 [48]. The geographic distance matrix was constructed in Geographic Distance Matrix Generator v.1.2.3 [49], while the genetic distance matrix was constructed in Mega v.6.06 [50].

### Demographic history

Mismatch distribution analysis was performed using the DnaSP v5.10 to develop figures and Arlequin 3.5 to calculate the values of the sum of squared deviations (SSD). The lack of significant differences in the SSD values indicated that the population assumed an expansion model.

The neutrality tests (Tajima’s *D* and Fu’s *F*s) were carried out on Arlequin 3.5 [51]. Negative Tajima’s *D* values suggest that a population has recently increased in size, whose results can tell the bottlenecks, selective effects, population expansion, or heterogeneity of mutation rates of *O. japonicus*.

### Biogeographic analysis

The statistical dispersal-vicariance analysis (S-DIVA) was implemented to infer ancestral distribution using RASP 3.2 [52]. The trees previously constructed by MrBayes were used as the input data. The geographical distribution of the species was categorized into five areas: (A) Sichuan province, China; (B) Zhejiang province, China; (C) Hubei province, China; (D) Hunan province, China; and (E) Guizhou province, China.

### Ecological niche modeling (ENM)

19 bioclimatic variables at a resolution of 2.5 arc minutes were obtained from the WorldClim website (available at http://www.worldclim.org/download), and community climate system model version 4 (CCSM4) model simulation was selected in Last inter-glacial (LIG), Last Glacial Maximum (LGM) and Mid Holocene (MID) periods. The current distribution information for *O. japonicus* was obtained from major databases and information systems, including the Global Biodiversity Information Facility (GBIF; http://www.gbif.org/) and the Chinese Virtual Herbarium (http://www.cvh.ac.cn). After a pairwise correlation analysis using SPSS, we selected a smaller set of nine relatively uncorrelated variables (r ≤ |0.8|): mean diurnal range (bio2), isothermality (bio3), temperature Seasonality (bio4), mean temperature of the wettest quarter (bio8), mean temperature of warmest quarter (bio10), mean temperature of coldest quarter (bio11), precipitation seasonality (bio15), precipitation of wettest quarter (bio16), and precipitation of driest quarter (bio17). Replicate runs (10) of MaxEnt using the “bootstrap” method were performed to ensure reliable results. Model performance was assessed using the area under the curve (AUC) of the receiver operating characteristic plot, with 25% of the localities randomly selected to test the model. Graphics for each predicted species distribution model were drawn using S-DIVA.

## Electronic supplementary material

Below is the link to the electronic supplementary material.

Supplementary Material 1

## Data Availability

The datasets generated and analysed during the current study are available in GenBank (accession numbers: OP785779-OP786045; OP786046-OP786312; OP786313-OP786579; OP786580 - OP786846).

## List of Abbreviations

cpDNA: chloroplast DNA
H: the number of haplotypes
*H*_d_: haplotype diversity
*π*: nucleotide diversity
*h*_S_: the average within-population gene diversity
*h*_T_: total gene diversity
*G*_ST_: the level of population differentiation at the species level
*V*_T_: the geographical total haplotype diversity
*V*_S_: geographical average haplotype diversity
*N*_ST_: an estimate of population subdivisions for phylogenetically ordered alleles
SSD: the sum of squared derivations
S-DIVA: the statistical dispersal-vicariance analysis
CCSM4: community climate system model version 4
LIG: last inter-glacial
LGM: last glacial maximum
MID: mid Holocene
GBIF: global biodiversity information facility
AUC: the area under the curve
*N*_m_: gene flow
AMOVA: analyses of molecular variance
RASP: reconstruct ancestral state in phylogenies
GRH: glacial refugium hypothesis
*F*_ST_: Genetic differentiation among populations
*F*_SC_: genetic differentiation among population within groups
*F*_CT_: Genetic differentiation between groups.
