# Supplementary material for: Phylogeography of cultivated and wild ophiopogon japonicus based on chloroplast DNA: exploration of the origin and sustainable cultivation
Source: BMC Plant Biol. 2023 May 8;23:242. doi: 10.1186/s12870-023-04247-2 (PMC10165772; doi:10.1186/s12870-023-04247-2)
Supplement: Supplementary file 1 — Supplementary Material 1 [file 12870_2023_4247_MOESM1_ESM.docx]

#### Table 1 Variable sites of the aligned sequences of 4 cpDNA in nineteen haplotypes (H1-H19) of *O. japonicus*

| Haplotype | | H20 | H1 | H2 | H3 | H4 | H5 | H6 | H7 | H8 | H9 | H10 | H11 | H12 | H13 | H14 | H15 | H16 | H17 | H18 | H19 |
| --- | --- | --- | --- | --- | --- | --- | --- | --- | --- | --- | --- | --- | --- | --- | --- | --- | --- | --- | --- | --- | --- |
| cpDNA | 59 | A | . | . | . | . | . | . | . | . | . | . | . | . | . | . | . | . | . | . | - |
|  | 60 | T | . | . | . | . | . | . | . | . | . | . | . | . | . | . | . | . | . | . | - |
|  | 61 | A | . | . | . | . | . | . | . | . | . | . | . | . | . | . | . | . | . | . | - |
|  | 62 | T | . | . | . | . | . | . | . | . | . | . | . | . | . | . | . | . | . | . | - |
|  | 63 | T | . | . | . | . | . | . | . | . | . | . | . | . | . | . | . | . | . | . | - |
|  | 192 | T | . | . | . | . | . | . | . | . | . | . | . | . | . | . | . | . | A | . | . |
|  | 204 | T | C | C | . | . | C | C | C | . | . | . | . | . | . | . | . | . | . | . | . |
|  | 309 | A | . | . | . | . | . | . | . | . | . | . | . | - | . | . | . | . | . | . | . |
|  | 404 | G | . | . | . | . | . | . | . | . | A | . | . | . | . | . | . | . | . | . | . |
|  | 545 | G | . | . | A | A | . | . | . | . | . | . | . | . | . | . | . | . | . | . | . |
|  | 666 | T | . | . | . | . | . | . | - | . | . | . | . | . | . | . | . | . | . | . | . |
|  | 667 | A | . | . | . | . | . | . | - | . | . | . | . | . | . | . | . | . | . | . | . |
|  | 668 | T | . | . | . | . | . | . | - | . | . | . | . | . | . | . | . | . | . | . | . |
|  | 669 | T | . | . | . | . | . | . | - | . | . | . | . | . | . | . | . | . | . | . | . |
|  | 670 | T | . | . | . | . | . | . | - | . | . | . | . | . | . | . | . | . | . | . | . |
|  | 671 | T | . | . | . | . | . | . | - | . | . | . | . | . | . | . | . | . | . | . | . |
|  | 672 | C | . | . | . | . | . | . | - | . | . | . | . | . | . | . | . | . | . | . | . |
|  | 673 | G | . | . | . | . | . | . | - | . | . | . | . | . | . | . | . | . | . | . | . |
|  | 674 | A | . | . | . | . | . | . | - | . | . | . | . | . | . | . | . | . | . | . | . |
|  | 675 | T | . | . | . | . | . | . | - | . | . | . | . | . | . | . | . | . | . | . | . |
|  | 676 | T | . | . | . | . | . | . | - | . | . | . | . | . | . | . | . | . | . | . | . |
|  | 677 | C | . | . | . | . | . | . | - | . | . | . | . | . | . | . | . | . | . | . | . |
|  | 678 | A | . | . | . | . | . | . | - | . | . | . | . | . | . | . | . | . | . | . | . |
|  | 679 | G | . | . | . | . | . | . | - | . | . | . | . | . | . | . | . | . | . | . | . |
|  | 723 | A | . | - | . | . | . | . | . | . | . | . | . | . | . | . | . | . | . | . | . |
|  | 724 | G | . | - | . | . | . | . | . | . | . | . | . | . | . | . | . | . | . | . | . |
|  | 725 | C | . | - | . | . | . | . | . | . | . | . | . | . | . | . | . | . | . | . | . |
|  | 726 | A | . | - | . | . | . | . | . | . | . | . | . | . | . | . | . | . | . | . | . |
|  | 727 | G | . | - | . | . | . | . | . | . | . | . | . | . | . | . | . | . | . | . | . |
|  | 728 | G | . | - | . | . | . | . | . | . | . | . | . | . | . | . | . | . | . | . | . |
|  | 729 | A | . | - | . | . | . | . | . | . | . | . | . | . | . | . | . | . | . | . | . |
|  | 756 | C | T | T | T | T | T | T | T | T | T | . | . | . | T | T | T | T | T | . | . |
|  | 758 | T | . | . | . | . | . | . | . | - | - | . | . | . | . | . | . | . | . | . | . |
|  | 759 | A | . | . | . | . | . | . | . | - | - | . | . | . | . | . | . | . | . | . | . |
|  | 760 | T | . | . | . | . | . | . | . | - | - | . | . | . | . | . | . | . | . | . | . |
|  | 761 | T | . | . | . | . | . | . | . | - | - | . | . | . | . | . | . | . | . | . | . |
|  | 762 | T | . | . | . | . | . | . | . | - | - | . | . | . | . | . | . | . | . | . | . |
|  | 763 | A | . | . | . | . | . | . | . | - | - | . | . | . | . | . | . | . | . | . | . |
|  | 764 | T | . | . | . | . | . | . | . | - | - | . | . | . | . | . | . | . | . | . | . |
|  | 765 | T | . | . | . | . | . | . | . | - | - | . | . | . | . | . | . | . | . | . | . |
|  | 766 | T | . | . | . | . | . | . | . | - | - | . | . | . | . | . | . | . | . | . | . |
|  | 794 | G | A | A | A | A | A | A | A | A | A | A | A | A | A | A | A | A | A | A | A |
|  | 907 | T | . | . | . | . | . | . | . | G | . | . | . | . | . | . | . | . | . | . | . |
|  | 917 | - | C | C | C | C | C | C | C | - | - | C | - | - | C | C | C | C | C | - | - |
|  | 918 | - | C | C | C | C | C | C | C | - | - | C | - | - | C | C | C | C | C | - | - |
|  | 919 | - | A | A | A | A | A | A | A | - | - | A | - | - | A | A | A | A | A | - | - |
|  | 920 | - | T | T | T | T | T | T | T | - | - | T | - | - | T | T | T | T | T | - | - |
|  | 921 | - | T | T | T | T | T | T | T | - | - | T | - | - | T | T | T | T | T | - | - |
|  | 949 | - | - | - | - | - | - | - | - | - | - | - | - | - | - | T | T | T | T | - | - |
|  | 950 | - | - | - | - | - | - | - | - | - | - | - | - | - | - | T | T | T | T | - | - |
|  | 951 | - | - | - | - | - | - | - | - | - | - | - | - | - | - | A | A | A | A | - | - |
|  | 952 | - | - | - | - | - | - | - | - | - | - | - | - | - | - | A | A | A | A | - | - |
|  | 953 | - | - | - | - | - | - | - | - | - | - | - | - | - | - | A | A | A | A | - | - |
|  | 954 | - | - | - | - | - | - | - | - | - | - | - | - | - | - | T | T | T | T | - | - |
|  | 955 | - | - | - | - | - | - | - | - | - | - | - | - | - | - | A | A | A | A | - | - |
|  | 956 | - | - | - | - | - | - | - | - | - | - | - | - | - | - | G | G | G | G | - | - |
|  | 957 | - | - | - | - | - | - | - | - | - | - | - | - | - | - | A | A | A | A | - | - |
|  | 958 | - | - | - | - | - | - | - | - | - | - | - | - | - | - | T | T | T | T | - | - |
|  | 959 | - | - | - | - | - | - | - | - | - | - | - | - | - | - | G | G | G | G | - | - |
|  | 960 | - | - | - | - | - | - | - | - | - | - | - | - | - | - | T | T | T | T | - | - |
|  | 961 | - | - | - | - | - | - | - | - | - | - | - | - | - | - | G | G | G | G | - | - |
|  | 962 | - | - | - | - | - | - | - | - | - | - | - | - | - | - | T | T | T | T | - | - |
|  | 963 | - | - | - | - | - | - | - | - | - | - | - | - | - | - | A | A | A | A | - | - |
|  | 964 | - | - | - | - | - | - | - | - | - | - | - | - | - | - | T | T | T | T | - | - |
|  | 965 | - | - | - | - | - | - | - | - | - | - | - | - | - | - | C | C | C | C | - | - |
|  | 966 | - | - | - | - | - | - | - | - | - | - | - | - | - | - | T | T | T | T | - | - |
|  | 967 | - | - | - | - | - | - | - | - | - | - | - | - | - | - | G | G | G | G | - | - |
|  | 968 | - | - | - | - | - | - | - | - | - | - | - | - | - | - | T | T | T | T | - | - |
|  | 1055 | G | A | A | . | . | . | A | . | . | . | A | . | . | A | . | . | . | . | . | . |
|  | 1184 | G | A | A | A | A | A | A | A | . | . | . | . | . | A | A | A | A | A | . | . |
|  | 1678 | C | T | T | . | . | . | . | . | . | . | . | . | . | . | . | . | . | . | . | . |
|  | 1733 | C | A | A | A | A | A | A | A | . | . | A | . | . | A | A | A | A | A | . | . |
|  | 1789 | G | . | . | . | . | . | . | . | . | . | . | . | . | . | A | A | A | A | . | . |
|  | 1844 | - | - | - | A | - | A | - | A | - | - | - | - | - | - | A | A | - | A | - | - |
|  | 1845 | - | - | - | T | - | T | - | T | - | - | - | - | - | - | T | T | - | T | - | - |
|  | 1846 | - | - | - | T | - | T | - | T | - | - | - | - | - | - | T | T | - | T | - | - |
|  | 1847 | - | - | - | T | - | T | - | T | - | - | - | - | - | - | T | T | - | T | - | - |
|  | 1848 | - | - | - | T | - | T | - | T | - | - | - | - | - | - | T | T | - | T | - | - |
|  | 1849 | - | - | - | A | - | A | - | A | - | - | - | - | - | - | A | A | - | A | - | - |
|  | 1850 | - | - | - | A | - | A | - | A | - | - | - | - | - | - | A | A | - | A | - | - |
|  | 1851 | - | - | - | A | - | A | - | A | - | - | - | - | - | - | A | A | - | A | - | - |
|  | 1852 | - | - | - | T | - | T | - | T | - | - | - | - | - | - | T | T | - | T | - | - |
|  | 1853 | - | - | - | T | - | C | - | C | - | - | - | - | - | - | C | C | - | C | - | - |
|  | 1854 | - | - | - | C | - | C | - | C | - | - | - | - | - | - | C | C | - | C | - | - |
|  | 1855 | - | - | - | T | - | T | - | T | - | - | - | - | - | - | T | T | - | T | - | - |
|  | 1856 | - | - | - | T | - | T | - | T | - | - | - | - | - | - | T | T | - | T | - | - |
|  | 1857 | - | - | - | C | - | C | - | C | - | - | - | - | - | - | C | C | - | C | - | - |
|  | 1858 | - | - | - | C | - | C | - | C | - | - | - | - | - | - | C | C | - | C | - | - |
|  | 1859 | - | - | - | A | - | A | - | A | - | - | - | - | - | - | A | A | - | A | - | - |
|  | 1860 | - | - | - | G | - | G | - | G | - | - | - | - | - | - | G | G | - | G | - | - |
|  | 1866 | A | . | . | . | . | . | . | . | . | . | . | . | . | . | . | . | . | . | C | C |
|  | 1871 | C | . | . | . | . | . | . | . | . | . | . | T | T | . | . | . | . | . | T | T |
|  | 1909 | A | - | - | - | - | - | - | - | . | . | - | . | . | - | - | - | - | - | . | . |
|  | 1910 | T | - | - | - | - | - | - | - | . | . | - | . | . | - | - | - | - | - | . | . |
|  | 1911 | A | - | - | - | - | - | - | - | . | . | - | . | . | - | - | - | - | - | . | . |
|  | 1912 | T | - | - | - | - | - | - | - | . | . | - | . | . | - | - | - | - | - | . | . |
|  | 1913 | T | - | - | - | - | - | - | - | . | . | - | . | . | - | - | - | - | - | . | . |
|  | 1914 | A | - | - | - | - | - | - | - | . | . | - | . | . | - | - | - | - | - | . | . |
|  | 1951 | A | . | . | . | . | . | . | . | . | . | . | . | . | . | . | G | . | . | . | . |
|  | 2169 | T | . | . | . | . | . | . | . | . | . | . | . | . | . | . | . | - | . | . | . |
|  | 2170 | C | . | . | . | . | . | . | . | . | . | . | . | . | . | . | . | - | . | . | . |
|  | 2171 | T | . | . | . | . | . | . | . | . | . | . | . | . | . | . | . | - | . | . | . |
|  | 2172 | T | . | . | . | . | . | . | . | . | . | . | . | . | . | . | . | - | . | . | . |
|  | 2173 | T | . | . | . | . | . | . | . | . | . | . | . | . | . | . | . | - | . | . | . |
|  | 2174 | C | . | . | . | . | . | . | . | . | . | . | . | . | . | . | . | - | . | . | . |
|  | 2175 | A | . | . | . | . | . | . | . | . | . | . | . | . | . | . | . | - | . | . | . |
|  | 2176 | A | . | . | . | . | . | . | . | . | . | . | . | . | . | . | . | - | . | . | . |
|  | 2177 | G | . | . | . | . | . | . | . | . | . | . | . | . | . | . | . | - | . | . | . |

Notes: All sequences are compared to the reference Hap20. ‘‘-’’ in sequences denote absence.
